# Spatial pattern formation facilitates eradication of infectious diseases

**DOI:** 10.1111/j.1365-2664.2007.01439.x

**Published:** 2008-04-01

**Authors:** Dirk Eisinger, Hans-Hermann Thulke

**Affiliations:** UFZ–Helmholtz Centre for Environmental Research UFZ, Department of Ecological Modelling (OESA) Permoserstr. 15, 04318 Leipzig, Germany

**Keywords:** cost–benefit, individual-based, management target, pattern-orientated modelling, rabies, spatial heterogeneity, vaccination

## Abstract

1. Control of animal-born diseases is a major challenge faced by applied ecologists and public health managers. To improve cost-effectiveness, the effort required to control such pathogens needs to be predicted as accurately as possible. In this context, we reviewed the anti-rabies vaccination schemes applied around the world during the past 25 years.
2. We contrasted predictions from classic approaches based on theoretical population ecology (which governs rabies control to date) with a newly developed individual-based model. Our spatially explicit approach allowed for the reproduction of pattern formation emerging from a pathogen's spread through its host population.
3. We suggest that a much lower management effort could eliminate the disease than that currently in operation. This is supported by empirical evidence from historic field data. Adapting control measures to the new prediction would save one-third of resources in future control programmes.
4. The reason for the lower prediction is the spatial structure formed by spreading infections in spatially arranged host populations. It is not the result of technical differences between models.
5. *Synthesis and applications.* For diseases predominantly transmitted by neighbourhood interaction, our findings suggest that the emergence of spatial structures facilitates eradication. This may have substantial implications for the cost-effectiveness of existing disease management schemes, and suggests that when planning management strategies consideration must be given to methods that reflect the spatial nature of the pathogen–host system.

## Introduction

**Fig. 1:**
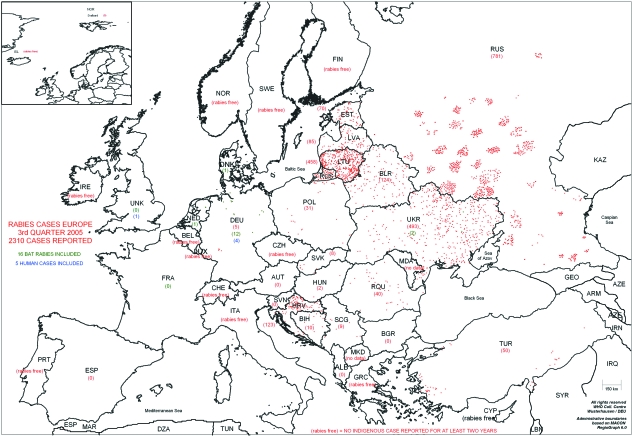
Confirmed cases of rabies in central Europe in 2005, second quarter (red dots). After a large-scale vaccination programme, central Europe is nearly free of sylvatic rabies, whereas it remains an issue in eastern Europe. Data provided by the WHO Collaborating Centre for Rabies Surveillance and Research in Europe, Wusterhausen, Germany.

Throughout the world 50–100 children are estimated to die from rabies infection every day (WHO 2005). Wildlife rabies poses a threat both to humans and to livestock in large parts of eastern Europe and the Americas (European Commission 2002; WHO 2005; Fig. 1). In the 1980s, large-scale oral vaccination campaigns were first used to fight the rabies epidemic successfully in foxes *Vulpes vulpes* (Stöhr & Meslin 1996; Vos *et al*. 2000). Nowadays, fixed-wing aircraft deliver vaccine-filled bait pieces, recording every drop with GPS precision (Breitenmoser & Müller 1997). However, there is still a debate on how many baits per unit area should be administered (WHO 2005), what percentage of the fox population needs to be immunized to ensure control success (Anderson 1986) and whether there is a threshold involved (Lloyd-Smith *et al*. 2005). In 1981, a population model predicted the threshold level to be about 70% for central European densities of about 3 foxes km^−2^ (Fig. 5 in Anderson *et al*. 1981). Subsequently, large-scale and long-term oral vaccination programmes in Europe were prepared to implement this target level following WHO/OIE guidelines (WHO 1992). Thus, for many years and over thousands of square kilometres, 18–20 baits km^−2^ and more have been administered to obtain a population level immunity above 70% (European Commission 2002).

However, empirical evidence indicates that, in regions with less then 70% immunization coverage, rabies has still been eliminated (Bugnon *et al*. 2004). As funding remains an issue for disease management, we need to explore any possibility of improving the cost–benefit ratio of control schemes (Meltzer & Rupprecht 1998). In this study, we re-analysed the management target of 70%, considering the ecological and epidemiological features of the fox–rabies system.

Our analysis was based on a spatially explicit individual-based simulation model. It was developed over a 10-year period and repeatedly validated against observed patterns of the fox rabies epidemic in Europe (Thulke *et al*. 1999a; Grimm *et al*. 2005). These patterns included the wave pattern and precursory epidemic foci (Jeltsch *et al*. 1997), residual epidemic foci after vaccination (Tischendorf *et al*. 1998) and the effects of an non-homogeneous bait distribution caused by aerial bait delivery (Thulke *et al*. 1999b). Additionally, the model was applied successfully to different aspects of large-scale vaccination programmes and post-vaccination emergency planning (Thulke *et al*. 2000; Eisinger *et al*. 2005).

**Fig. 2:**
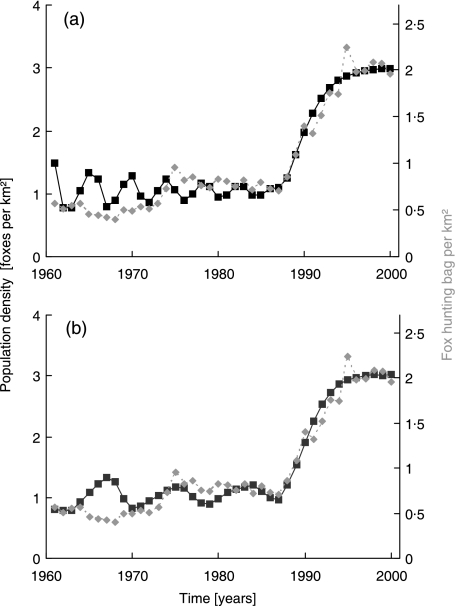
Hunting bag of Germany compared with fox population densities of (a) the population model (Anderson *et al*. 1981) (*K* = 3, *K_t_* = 1) and (b) the simulation model. Hunting bag data (grey line) are given relative to hunted area (Bellebaum 2003). The figures show population (left, model) and hunting density (right, data) with epidemic rabies and after successful eradication. Vaccination in the models started in 1988. For reference scaling, the disease-free situation was used, i.e. a spring density of 3 foxes km^−2^ corresponds to 2 hunted foxes throughout the year. The population reduction by the disease as well as the rate of recovery after the onset of vaccination resulted independently from both modelling approaches.

For a quantitative comparison with the classic model from 1981 (Anderson *et al*. 1981), both studies had to represent the same ecological system. Therefore we applied the logic of pattern-oriented modelling (Grimm *et al*. 2005). As the multilevel pattern, we used Germany's hunting bag data from the last 40 years, which covers periods with and without the roaming rabies epidemic and the running vaccination programme (Bellebaum 2003). Because these patterns captured the overall ecological dynamics of our study system, any realistic model of the system should reproduce these patterns before it can be used for predictions. Figure 2 shows the comparison of the hunting bag data with time series produced by the population model and the simulation model. Both models worked equally well. Thus we addressed what level of immunization is required in either of these models to eradicate rabies by mass vaccination.

## Materials and methods

We applied a time-discrete, spatially explicit, individual-based modelling approach with a 1-week time step (DeAngelis & Gross 1992; Durrett 1995; Grimm & Railsback 2005). The spatial unit was the home range of a fox group (i.e. spatial group; Macdonald 1983) represented by a quadratic cell. Conceptually, we did not approximate the natural shape and extent of home ranges by the cell but used it as a technical representation. The total extent of the model area covered 256 × 256 cells. Within the group, foxes were represented as individuals. In the following we first describe the main aspects of the model: fox population dynamics, rabies transmission and bait distribution. We name the model rules with their literature-based reference parameterization (see Table S1 in the supplementary material). Secondly, we define successful immunization, introduce the population model used in the literature for predicting the minimum immunization level, explain how the models are aligned, and finally determine experimental variants of the simulation model.

### fox population dynamics

Each cell comprises a group of age-classified individual foxes (juvenile, adult; Macdonald 1983; Cavallini 1996). After mating, rural fox families contain on average two to three adults (i.e. one male and one to two females; Niewold 1980; Cavallini 1996), which, together with floater foxes (Vos 2003), results in an observed population density of about 3 adults km^−2^. In the model, this pattern is translated by assuming a maximum of 5 adults cell^−1^ (Goszczynski 2002) together with literature values of mortality without rabies (i.e. 1·3% week^−1^ in adults and 2·7% in juveniles before dispersal) and the dispersal process (Ansorge 1990; Woollard & Harris 1990; Stiebling 2000). Reproduction is scheduled in the first week of April. All fox groups produce on average 5·5 cubs (normal distribution limited between 0 and 13, SD 1·5; Lloyd 1980; Ansorge 1990; Goretzki *et al*. 1997; Stiebling 2000). Fox groups of exactly one individual reproduce with 50% probability, reflecting a sex ratio of 1:1 and polygamous mating behaviour, which leaves hardly any female unmated (Niewold 1980). With these population dynamics, on average 3·5 juveniles survive to disperse in populated cells. The dispersal occurs from October to November (Storm & Montgomery 1975; Lloyd 1980). During that phase, one-eighth of all cells are selected at random per week. From each selected cell, all juveniles move consecutively. The dispersing individual is randomly assigned with a main direction (Storm & Montgomery 1975; Storm *et al*. 1976) corresponding to one of the eight neighbouring cells. In each dispersal step the individual passes one cell, i.e. group home range. To do so it continues in the main direction with 50% probability or deviates by one cell to the left or to the right with 25% probability, respectively (Jeltsch *et al*. 1997). The probability of settling (PSettle) increases with the number of cells travelled (*F*_Pathlenght_; Trewhella, Harris & McAllister 1988) but decreases with the number of adult foxes encountered in the target cell (*F*_Crowding_):
eqn 1
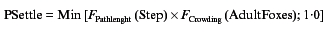

eqn 2
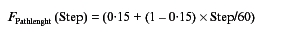

eqn 3
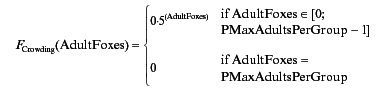


During each step we assume a mortality of 1·5%, which, by running the model with the reference density, results in 22% overall mortality of dispersed foxes, as reported from the field (Woollard & Harris 1990). The dispersal of one individual is limited to 100 steps (Jensen 1973; Steck & Wandeler 1980) and an individual that cannot settle eventually dies, which is hardly ever reached in simulations. After dispersal juveniles are treated as adults. We tested the model correctness with empirical observations (i.e. patterns) by resampling respective features from the model (Grimm 2002). For example, for a spring density of three foxes, the average dispersal distance resulting from the algorithm corresponds to about 14·4 km (median 11·5 km). In comparison with the literature (Jensen 1973), the reported pattern of cumulative distance distribution re-emerged from the model algorithm (Eisinger *et al*. 2005). Because of population turnover, the actual population size varies throughout the year therefore any density reference refers to the situation before reproduction (i.e. first week of March) and scales per cell.

### rabies transmission

**Fig. 3:**
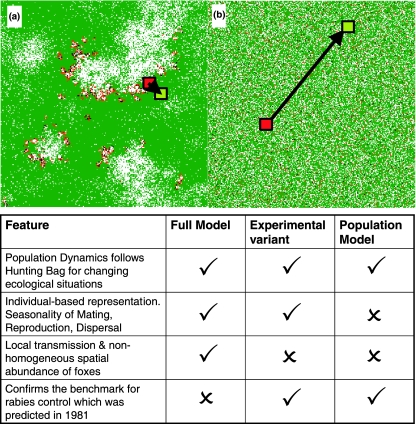
Snapshots of the simulation model, (a) the original and (b) the experimental variant without spatially structuring processes. The snapshot of the full version of our model shows the typical wave pattern and epidemic foci as produced by the predominant neighbourhood infection. Local transmission is depicted by an arrow from the infected fox group (red square) to the susceptible fox group (green square). As a result of global transmission the experimental variant without spatially structuring processes shows completely mixed infected and susceptible foxes, effectively meaning that every infected fox might infect any susceptible fox, however distant they may be (indicated by the long arrow).

Each fox has a disease state (susceptible, infected, infectious or immune). If infection is introduced in a cell through neighbourhood contact, one adult fox is randomly selected. If its state is susceptible, it changes to infected. The infected fox incubates and gets infectious after a time period that is drawn randomly from a negative exponential distribution, with a minimum of 2 weeks and an effective mean of 3·5 weeks (Reichert 1989; Barrat & Aubert 1993). During the following infectious period of 1 week (Macdonald & Bacon 1982), a fox can transmit the disease using the approach of infection communities (Thulke *et al*. 1999a) or group infection rate (Macdonald & Bacon 1982). If there is at least one infectious fox in a cell, all other susceptible foxes within the cell become infected in intragroup contacts (Macdonald & Bacon 1982; Vos 2003). Additionally, the eight neighbouring cells have a probability of 16% of getting infected in neighbourhood contacts (adjusted to hunting bag pattern) and in January and February any neighbouring cell within a distance of up to 3 cells may be infected, with a probability of 0·16^1^, 0·16^2^ and 0·16^3^, respectively, because of mating contacts (Storm & Montgomery 1975; Macdonald & Bacon 1982; Tischendorf *et al*. 1998; Vos 2003). There are hardly any infections during dispersal (Storm & Montgomery 1975; Niewold 1980; Vos 2003) but juvenile foxes dispersing during their incubation period will cause standard transmission after settlement (Vos 2003). For a snapshot of the resulting dynamic, see Fig. 3a. Different patterns re-emerge out of the set of model rules, for example the seasonal rabies peaks caused by mating activities and dispersal (Toma & Andral 1977; Bögel *et al*. 1981; Vos 2003) and the focal spatial pattern of the advancing rabies epidemic (Sayers *et al*. 1985; Jeltsch *et al*. 1997; Thulke *et al*. 1999a).

### distribution of baits

We apply standard biannual campaigns, with one vaccination event in the first week of April and one in the second week of September (European Commission 2002). We approximate the non-equal assignment of baits to fox groups (Breitenmoser & Müller 1997) by simulating the distribution of effective bait numbers on the ground for standard aerial delivery (Thulke *et al*. 2004). Additionally, baits are assumed to be lost with 80% probability, i.e. because of non-target competitors (Vos *et al*. 2000) and not found or only partly consumed baits (Müller 1998; Vos *et al*. 2000; European Commission 2002). The remaining baits are distributed randomly to the individuals in a cell. The susceptible foxes permanently turn immune 2 weeks after receiving at least one piece of bait (Barrat & Aubert 1993; Müller 1998). Following the pattern-oriented paradigm, we validated the emergent outcome of the vaccination algorithm against empirical patterns found in the literature (Grimm 2002), i.e. immunization rate per number of campaigns (Barrat & Aubert 1993; Vos *et al*. 2000; Hostnik *et al*. 2006).

We were interested in the effect of changing the immunization target in terms of the necessary control effort. The respective relationship is constructed by resampling the simulation model for a reference spring population density of 3 foxes km^−2^. Technically we perform 100 simulation runs for any integer bait density value from 4 to 30 and record the resulting immunization level from the saturated situation (i.e. after 5 years, ranging between 30% and 85%). Finally, we pair available absolute surplus values in immunization level (i.e. between 0% and 20%) with corresponding relative change in bait density (i.e. 0–100%).

### definition of eradication

We use the same definition for eradication as in the original work of Anderson *et al*. (1981), namely a negative growth rate of rabies even if the fox population is at the maximum density (the disease-free fox density). In the simulation model we determine this by starting with a fox population at the disease-free density with the desired level of population immunization and 100 infected foxes randomly distributed on the grid. If rabies has a negative growth rate under these conditions, rabies cannot establish in the population. Thus, if rabies is unable to invade the immunized fox population in any of 1000 repetitions of a simulation scenario, we equate this to 100% eradication. With respect to practical management, we additionally applied a more feasible criterion where the full history of the management was followed up explicitly in the simulation (i.e. starting from an epidemic situation) and control was defined successful when, after 4 years of repeated vaccination campaigns, rabies was eradicated with 95% probability (Aubert 1994; Thulke *et al*. 2000).

### the population model

We compare the simulation model with the population model detailed in Anderson *et al*. (1981), which uses three differential equations to represent the susceptible, incubating and infectious cohorts of the fox population (see Appendix S1 in the supplementary material). Infections are modelled proportional (i.e. transmission parameter) to the susceptible fox density times the infectious fox density. This model predicts eradication of rabies if the percentage of protected fox population is higher than 1 –*K_t_*/*K* (*K* = the disease free density, *K_t_* = the minimum fox density that allows the disease to persist depending on the transmission parameter). We use the same parameterization as in the original 1981 work, specifically *K_t_* = 1 fox km^−2^.

### model alignment

In order to compare the prediction of two models they must represent the same ecological system. By modifying, for example, disease transmission dynamics (i.e. transmission parameter), host reproductive ecology (i.e. birth and mortality) and habitat capacity (i.e. density without disease) in a model, the represented ecological system is assumed to have changed. Thus it would be unsurprising if predictions of management efficacy varied accordingly. Therefore the alignment of both models is a prerequisite of our analysis. We use the pattern of long-term hunting bag data from the last 40 years (Bellebaum 2003) to align our candidate models. Hunting bags are notoriously unreliable, as changes in behaviour and legislation might well be visible. However, as the data are pooled over all Germany, local spatial or temporal effects are removed. The strength of the multilevel pattern is that all periods of the rabies epidemic and its control are inherently covered. We have fingerprints of the fox–rabies dynamics (i) with disease (epidemiological processes in the model), (ii) without disease (ecological processes) and (iii) the rate of recovery (interaction of processes). Technically, the level of foxes shot after disease eradication is associated with the size of the fox population without disease (i.e. disease-free density *K* in Anderson *et al*. 1981); the relative reduction of the number of foxes shot before any control gives us the figure of how transmission efficiency reduces the fox population (i.e. resulting in *K_t_* in Anderson *et al*. 1981). The slope of the increase in hunting bag after control pinpoints the natural speed of eradication and population recovery that is used to test the structural realism of the models (Wiegand *et al*. 2003; Grimm *et al*. 2005).

### the experimental model variants

The experimental variants of the simulation model result from the manipulation of particular rules. Thus relevant biological details of the system are artificially removed from the model in order to analyse the behaviour of the remaining ‘incomplete’ representation of the fox–rabies system. For example, instead of seasonal reproduction as reported in literature, we schedule some birth event every week of the year, thereby mimicking instantaneous reproduction. Similarly, in the experimental variant without spatially structuring processes, rabies transmission acts globally (Fig. 3a,b) and vaccination is assumed to cover the fox population homogeneously (see Table S2 in the supplementary material). Technically, we multiply the overall densities of infected foxes, the number of susceptible foxes and the transmission parameter. The resulting density is recalculated into abundance of newly incubating foxes, which are then identified with randomly selected susceptible individuals. The foxes are completely mixed after each time-step by putting them randomly into the fox groups, but retaining the density distribution map. To remove the spatial effect of the non-random bait distribution, we perform vaccination by keeping the number of immune foxes at the level corresponding to the desired population immunity.

## Results

**Fig. 4:**
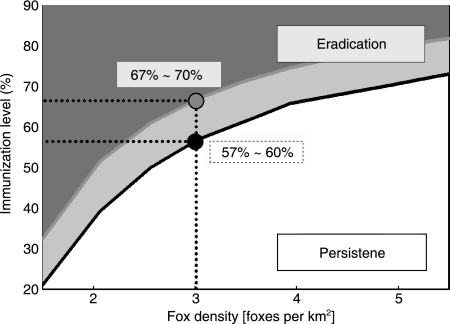
Required immunization level necessary for successful elimination as predicted by the population model (grey line; cf. Fig. 5 in Anderson *et al*. 1981) and the simulation model (black line) for different disease-free densities. Our model predicts a minimum level lower by about 10%. Specifically, with a disease-free density of 3 foxes km^−2^, we found a threshold of about 57% in contrast to the 67% predicted by the population model. The stochastic simulation model also yields some chance of eradication in the area below the black line (even lower immunization level; see Fig. S1 in the supplementary material); however, here we show 100% eradication (i.e. all 1000 repetitions must have resulted in eradication).

For all population densities, the spatial simulation model generally needs around 10% lower immunization coverage to eradicate rabies compared with the population model (Fig. 4). Furthermore, the criterion applied to determine success (i.e. either negative growth rate or 95% within 4 years; see the Methods) does not influence the finding (see Fig. S1 in the supplementary material).

**Fig. 5:**
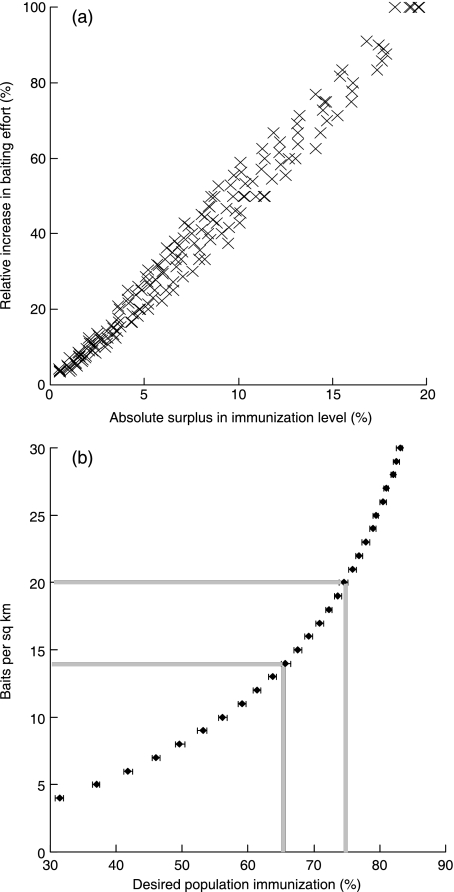
The relationship between desired immunization level and baiting effort in the model assuming a disease-free density of 3 foxes km^−2^. (a) The graph represents the relative increase in baiting effort if the target level of population immunization is increased. The linear relationship reveals a non-linear increase in absolute baiting effort per unit of target immunization level. This is the result of bait competitors and foxes consuming multiple baits. For example, applying a 10% higher management target increases baiting effort by 42%. (b) Adopting recent safe-side baiting practice from the field that targets a 75% immunization coverage (right vertical grey line) by applying 20 baits km^−2^ (upper horizontal line), we explored the relationship to find out what would result from a reduction in the target level by 10% (left vertical line) and came to 14 baits km^−2^ (lower horizontal line). Whiskers represent 5% and 95% percentiles from all repetitions.

In particular, with a disease-free density of 3 foxes km^−2^, we found a threshold of about 60%, in contrast with 70% derived from the population model. The obvious explanation for this systematic disagreement is a difference in modelling approach (i.e. stochastic vs. deterministic, individual-based vs. population-based, spatially explicit vs. non-spatial). To investigate this in more detail, we derived experimental variants of our simulation model. The experimental models mimic the methodological assumptions underlying the population model. Practically, this means that biological processes are systematically switched off or on. Several variants were tested and combined (Eisinger & Thulke 2005). Surprisingly, we only needed to switch off the biological features that account for the emergence of spatial patterns in the infection: local transmission and spatially heterogeneous vaccination (see the Methods; Fig. 3). This (and only this) variant of our model yielded exactly the same prediction as the analytical population model, again robust over the whole range of population density values. Because all models are aligned equally (Fig. 2), the lowered prediction we found is a result of the spatial pattern formation by biological processes responding to local neighbourhood differences.

The relationship between increased target immunization level and relative change in baiting effort was found to be linear but independent of the base line target level (Fig. 5a; limited to analysed immunization levels between 30% and 85%). Applying a management target that is 10% higher relates to a surplus in baiting effort of 42% or, vice versa, reducing the target by 10% corresponds with a reduction in baiting resources by one-third (Fig. 5a).

## Discussion

Our results suggest that rabies vaccination campaigns in the past utilized excessive baits. Those baits can be saved without jeopardizing success if the vaccination target is reduced by 10% of population immunity. The economic benefit of the reduction would be one-third of baiting resources. These resources could be used elsewhere; for example, in eastern Europe large-scale standard control schemes are constrained by legislation because of the enormous cost per unit area. Here the saved baits as the result of a lower target would allow for treatment of larger areas. Or savings could be budgeted to control rabies in urban areas. In Germany, rabies persisted longest in the large urban settlements in Hessia, where baits could not be distributed easily by fixed-wing aircraft (European Commission 2002). Thus bait distribution by helicopters and by hand was necessary, at additional cost. However, prior to improved resource utilization, the traditional benchmark has to be revised. Field estimates of population immunity below 70% are found in some control areas, in concordance with achieved elimination. Still today such findings are used to doubt the precision of the estimated immunity level rather then the appropriateness of the benchmark as a target itself.

### baiting effort

We do not equate the predicted success level of 57% immunized foxes to an absolute number of baits because field data on the relationship between baits distributed and immunization level achieved is still uncertain (Barrat & Aubert 1993; Selhorst & Müller 1999; Hostnik *et al*. 2006; T. Müller, unpublished data). In the literature this relationship is approximated by estimating seroprevalence (i.e. antibodies in serum indicating fox immunity) or bio-marker prevalence (i.e. OTC, a chemical added to the bait that marks the consumer for life). However, these population-based estimates suffer from sampling uncertainty and from well-known conflicting technical properties [e.g. 4/25 experimentally vaccinated foxes did not test serologically positive (immune) but they survived infection; T. Müller, personal communication]. Furthermore, the seroprevalence estimate of the Swiss control programme was found to range between 50% and 80% (Wandeler *et al*. 1988). Although field data do not provide a definite link between the number of baits giving a certain immunization level in the target population, we nevertheless can use our results to quantify the gain from a reduced target level within the existing framework of rabies control (Fig. 5b). At present, European baiting strategies apply 20 baits km^−2^ to meet the safe-side management target of a 75% immunization level. Our results suggest that a reduction of 10% in the target level (i.e. yielding a 65% immunization level) would require the application of only 14 baits km^−2^ (Fig. 5b). This is supported by data from Switzerland, where eradication was achieved using 12–15 baits km^−2^ in the 1980s (Wandeler *et al*. 1988).

### re-assessment of the traditional benchmark

Our analysis revealed that the existing benchmark for the level of rabies control is imprecise, yet it is used widely in management planning and as quality assurance. By taking into consideration the limited range of local transmission and non-uniform access to bait pieces by foxes, the target could be reduced by 10%. The inherently predictive nature of control planning requires a conservative standpoint, therefore we only considered simulations with zero failure risk when deriving Fig. 4(i.e. comparable to the approach of population models). In future, more risky strategies could be explored to investigate different economic scenarios; for example, our simulations resulted in rabies elimination in every second trial for immunization levels below 50% (see Fig. S1 in the supplementary material).

At present, the European Community does not co-fund national rabies control programmes that do not satisfy the benchmark. Fulfilling this demand is often difficult but, according to our results (and field evidence), it is not necessary for control success. Certain European vaccination programmes have succeeded in regions where less then 70% of foxes have been found to be immune. Unfortunately some of these data are not published (e.g. German Bundesland Sachsen carried out a population test in 1995–96 and estimated 32–64% immunity in adult foxes while rabies was successfully eradicated; T. Müller, personal communication; Baker *et al*. 2001) yet they may provide evidence of an imprecise benchmarking of control programmes.

Although control measures that reach the currently applied benchmark will be successful, the associated cost is unnecessary in light of the more precise target reported here. Cost-effectiveness is an issue in rabies control programmes, for example in eastern European areas characterized by roaming rabies (Fig. 1), small financial resources but huge target populations. With a single bait costing more than 1 euro, a reduction from 20 to 14 baits km^−2^ would save 15 million euro year^−1^ in rabies control in the frontier states (Belarus, Ukraine and the Baltic States; Fig. 1). By revising the current management benchmark, it would be possible to attain a realistic level of rabies control over a greater area (Selhorst, Thulke & Müller 2001; Bohrer *et al*. 2002).

### robustness of model prediction

There may be an effect of the particular grid structure of the model on the prediction of the spatial simulations (G. C. Smith, personal communication). To address this issue, the complete simulation was repeated with three (i.e. regular triangulated grid), four (i.e. von Neumann neighbourhood) and six (i.e. hexagonal grid; Suppo *et al*. 2000) neighbours. After applying the new versions of the model to the overall pattern of the hunting bag data, the predictions were found to be in complete agreement regarding the minimum immunization level required for rabies eradication (results not shown). Given that none of the constantly modelled interfox contact structures will correspond to home range assemblages in the field, we also assigned the contact neighbourhood for each fox group and each time step by chance. This reflects a random neighbourhood structure throughout the grid, which changes continuously because of competitive interaction between groups or stochastic removal because of mortality. Once again, there was complete agreement between the predicted levels of necessary immunity to wipe out the disease (results not shown). From these additional simulations, we conclude that the results we present here are qualitatively and quantitatively independent of the assumed number of potential contact neighbours for a fox group; therefore any bias in our prediction because of grid configuration is excluded.

### why does spatial clustering lead to a lower immunization level needed to control rabies?

Towards the end of a control programme, a small number of infected hosts is still observed in the fox population (demonstrated in the field by large-scale surveillance). Contrary to the assumption of homogeneous mixing, for these local remnants of rabies infection the probability of meeting a susceptible fox is reduced by the spatial structure that might place immune animals around them. This effect will be exacerbated by non-homogeneous bait coverage resulting in local areas of above-average immunization (i.e. 100%). The reciprocal situation (an infectious individual in a local neighbourhood with below-average immunity) is of no practical consequence because disease spread is local, hence many of the local foxes will already be infected (Thulke *et al*. 1999b). To represent these ecological features in a model, we have to consider explicitly the spatial arrangements of fox hosts. At present, this is not feasible in the analytic population model; hence the supporting effect of spatial structuring on the control outcome could be measured only with the spatially explicit simulation model.

### conclusion

It is widely acknowledged that spatially explicit consideration of disease transmission is necessary to model epidemics accurately (Levin & Durrett 1996; McCallum, Barlow & Hone 2001; Lloyd-Smith *et al*. 2005). We recognize that non-spatial population models might overestimate the minimum immunization level necessary for disease control. In particular this holds for diseases where the predominant mode of transmission is local, leading to a spatially structured epidemic and a non-random distribution of the susceptible host species. We recommend that target values for application of oral vaccines in disease control programmes should not be based solely on the prediction of population models. Our study supports the view that individual-based models of complex systems have wide application in quite different areas of ecological research (Grimm *et al*. 2005) and that they are ideally suited to improve targeting of disease control programmes in wildlife populations.

Finally, we demonstrate that the current anti-rabies control guidelines, with a target level of 70% immunized foxes, gives a wide margin of safety. Thus there is no value in increasing baiting density to improve rabies control (Frost *et al*. 1985; Cliquet *et al*. 2000; Hostnik *et al*. 2006). Other approaches need to be considered where the present level of control does not eliminate rabies from the host population.

## Supplementary material

The following supplementary material is available for this article.

Appendix S1 Short sketch of population model by Anderson et al. (1981)

Table S1 Parameters of the simulation model, default values and references

Table S2 Parameter substitutions in the experimental model compared with Table S1

Fig. S1 Probability of eradication for different control strategies.

This material is available as part of the online article from: http://www.blackwell-synergy.com/doi/full/10.1111/j.1365-2664.2007.01439.x (This link will take you to the article abstract.)
