# Supplementary material for: Spatial pattern formation facilitates eradication of infectious diseases
Source: J Appl Ecol. 2008 Apr 1;45(2):415–23. doi: 10.1111/j.1365-2664.2007.01439.x (PMC2326892; doi:10.1111/j.1365-2664.2007.01439.x)
Supplement: Appendix S1 — Short sketch of population model by Anderson et al. (1981) [file jpe0045-0415-SD1.doc]

**Supplementary Material**

**Spatial Pattern Formation Facilitates Eradication of Infectious Diseases**

Dirk Eisinger & Hans-Hermann Thulke

UFZ, Helmholtz Centre for Environmental Research - UFZ; Department of Ecological Modelling (OESA); Permoserstr. 15; 04318 Leipzig; Germany.

Email: rabies@ecoepi.de

**Content:**

**Appendix S1:** Sketch of the analytical model applied by Anderson *et al.* (1981) to predict minimum immunisation level in oral mass vaccination of foxes.

**Table S1.** Parameters of the simulation model, default values and reference.

**Table S2.** Parameter substitutions in the experimental model.

**Figure S1.** Probability of eradication for different control strategies.

**Appendix S1. The population model by Anderson *et al.* (1981).**

The model consists of three coupled differential equations:

dX/dt = rX – (b+γN)X – βXY

dI/dt = βXY – (σ+b+γN)I

dY/dt = σI – (α+b+γN)Y

with N, X, I and Y being the total, susceptible, incubating and infectious fox densities respectively. The model was developed to reflect rabies dynamics in the fox population and to assess feasibility of oral mass-vaccination as control strategy against the disease. Using differential equations the model is based on infinitesimal time-steps and infinitesimal changes in densities (i.e. fractions of individual). Population ecology is represented by continuous mortality (b), reproduction (r) and density-dependent mortality of dispersing juveniles (γ). Transmission is modelled as mass action (McCallum, Barlow & Hone 2001; Nokes & Anderson 1988), i.e. is determined by the product of the susceptible population times the infectious population times the transmission rate (β). Infectious course is modelled by average length of incubation (1/σ) and infectious period (1/ α). Vaccination is modelled as a linear reduction of the susceptible population by the level of vaccination. The percentage of infectious fox population asymptotically approaches zero in case of a negative growth rate of the rabies infection. Consequently the combat strategy of the disease was defined successful when resulting in a negative growth rate of rabies. The population model predicts for typical Central Europe density estimates of about 3 foxes per km², the vaccination of 70% of the fox population as sufficient for eradication (cf. Fig. 5 in original paper).

**Table S1.** Parameters of the simulation model, default values and reference.

| Parameter | Value | Reference |
| --- | --- | --- |
| Population Ecology | | |
| PMaxAdultsPerGroup | 4-9 ^1^ | Based on (Goszczynski 2002) |
| PWeeklyMortalityAdults | 0.53-1.69 % ^1^ | Based on (Stiebling 2000) |
| PWeeklyMortalityJuveniles | 1.02-3.42 % ^1^ | Based on (Stiebling 2000) |
| PLitterMeanSize | 5.5 | (Lloyd 1980; Goretzk*i et* al. 1997; Gortaza*r et* al. 2003) |
| PLitterStdDev | 1.5 | (Goretzk*i et* al. 1997) |
| Juveniles’ Dispersal (October-November) | | |
| PDispersalLengthOfDispersalPeriod | 8 weeks | (Storm & Montgomery 1975) |
| PDispersalProbabilityNotToLeave | 15 % | (Jensen 1973; Steck & Wandeler 1980; Goretzk*i et* al. 1997) |
| PDispersalIntrinsicMaxDistance^3^ | 60 steps | (Jeltsc*h et* al. 1997) |
| PDispersalMaximumDistance^3^ | 100 steps | (Jensen 1973; Steck & Wandeler 1980) |
| PDispersalMortalityPerStep | 1.5 % ^2^ | Adjusted to (Woollard & Harris 1990) |
| Rabies Epidemiology | | |
| PIncubationPeriodMean | 3.5 weeks | (Reichert 1989) |
| PIncubationPeriodMinimum | 2 weeks | (Reichert 1989) |
| PTransmissionProbabilityPerNeighbourGroup | 16.4 % ^4^ | Following (Müller 1995; Tischendor*f et* al. 1998) |
| PTransmissionBasicProbabilityMaiting | 16.4 % ^4^ | Following (Müller 1995; Tischendor*f et* al. 1998) |
| Management Strategy | | |
| PManagementArea | 256*256 km² |  |
| PBaitLoss | 80 % ^5^ |  |
| PManagementBaitDensity | 2-30 bpkm² ^1^ |  |

^1^ Set in accordance with population density or population immunity level required in simulation scenarios.

^2^ Adjusted to 22% dispersed foxes found dead for a population with about 3 foxes per sq km between mating period and reproduction (Woollard & Harris 1990).

^3^ PDispersalIntrinsicMaxDistance detemines the slope of PSettle; PDispersalMaximumDistance is the maximum number of steps during dispersal. Any individual which hasn’t settled is removed.

^4^ Adjusted to realize level of population reduction by the disease.

^5^ Adjusted to 75% immunization level at 20 baits per km².

**Table S2**. Parameter substitutions in the experimental model compared to Table 1.

| Parameter | Value | Value’s reasoning |
| --- | --- | --- |
| Rabies Epidemiology | | |
| PGlobalTransmissionRate  *replaces*  PTransmissionProbabilityPerNeighbourGroup  PTransmissionBasicProbabilityMaiting | 1.534 | Transmission parameter.  The value was adjusted to realize right level of population reduction by the disease. The actual number emerged and was identical to the value used in para-meterization of the classical model. |
| Management Strategy | | |
| PPopulationImmunization  *replaces*  PBaitLoss  PManagementBaitDensity | 20-80 % | Ad hoc level of homoge-neous vaccination in the whole host population |

**Figure S1.** **Probability of eradication for different control strategies.**

The graphs show the eradication probability for disease free density of 3 foxes per sq km. Starting with any target value for the immunisation coverage (left axis) horizontally the respective probability of eradication is given over time of control. (A) Test against growth rate of rabies. Equivalently to Anderson *et al*. (1981) we defined a negative growths rate as 100% eradication within 20 years. Hence, the lowest immunisation coverage to achieve eradication is 58%. (B) Eradication of an epidemic: With respect to practical management we tested a more feasible criterion where control was defined ‘successful’ when after 4 years of control rabies was eradicated with 95% probability which again requires about 58%.

#

# References

Anderson, R.M., Jackson, H. C., May, R. M. & Smith, A. D. M. (1981) Population dynamics of fox rabies in Europe. *Nature*, **289**, 765-770.

Goretzki, J., Ahrens, M., Stubbe, C., Tottewitz, F., Sparing, H. & Gleich, E. (1997) Zur Ökologie des Rotfuchses (*Vulpes vulpes* L.,1758) auf der Insel Rügen: Ergebnisse des Jungfuchsfanges und der Jungfuchsmarkierung. *Beiträge zur Jagd- und Wildforschung*, **22**, 187-199.

Gortazar, C., Ferreras, P., Villafuerte, R., Martin, M. & Blanco, J. C. (2003) Habitat related differences in age structure and reproductive parameters of red foxes. *Acta Theriologica*, **48**, 93-100.

Goszczynski, J. (2002) Home ranges in red fox: territoriality diminishes with increasing area. *Acta Theriologica*, **47**, 103-114.

Jeltsch, F., Müller, M. S., Grimm, V., Wissel, C. & Brandl, R. (1997) Pattern formation triggered by rare events: lessons from the spread of rabies. *Proceedings of the Royal Society of London Series B-Biological Sciences*, **264**, 495-503.

Jensen, B. (1973) Movements of red fox (Vulpes vulpes L.) in Denmark investigated by marking and recovery. *Danish Review of Game Biology*, **8**, 3-20.

Lloyd, H.G. (1980) *The red fox.* B.T.Batsford Ltd., London.

McCallum, H., Barlow, N. & Hone, J. (2001) How should pathogen transmission be modelled? *Trends in Ecology and Evolution*, **16**, 295-300.

Müller, M.S. (1995) *Ein gitterbasiertes Modell zur Tollwutausbreitung bei Füchsen (Vulpes vulpes).* Diploma thesis, University Marburg/Lahn, Germany.

Nokes, D.J. & Anderson, R. M. (1988) The use of mathematical models in the epidemiological studies of infectious diseases and in the design of mass immunization programs. *Epidemiology and Infection*, **101**, 1-20.

Reichert, H.-U. (1989) *Simulationsstudien zur Ausbreitung und Bekämpfung der Tollwut bei Füchsen mit einem stochastischen, räumlichen Modell.* PhD thesis, University Frankfurt am Main, Germany.

Steck, F. & Wandeler, A. (1980) The epidemiology of fox rabies in Europe. *Epidemiologic Reviews*, **2**, 72-96.

Stiebling, U. (2000) *Untersuchungen zur Habitatnutzung des Rotfuches, (Vulpes vulpes L., 1758), in der Agrarlandschaft als Grundlage für die Entwicklung von Strategien des Natur- und Artenschutzes sowie der Tierseuchenbekämpfung.* PhD thesis, Humboldt University Berlin, Germany.

Storm, G.L. & Montgomery, G. G. (1975) Dispersal and Social Contact among Red Foxes: Results from Telemetry and Computer Simulation. *The Wild Canids* (ed M.W. Fox), pp. 237-246. Van Nostrand Reinhold Co., New York.

Tischendorf, L., Thulke, H.-H., Staubach, C., Müller, M. S., Jeltsch, F., Goretzki, J., Selhorst, T., Müller, T., Schlüter, H. & Wissel, C. (1998) Chance and risk of controlling rabies in large-scale and long-term immunized fox populations. *Proceedings of the Royal Society of London Series B-Biological Sciences*, **265**, 839-846.

Woollard, T. & Harris, S. (1990) A behavioural comparison of dispersing and non-dispersing foxes (*Vulpes vulpes*) and an evaluation of some dispersal hypotheses. *Journal of Animal Ecology*, **59**, 709-722.
